# Nonparametric estimation of characteristics of the interspike interval distribution

**DOI:** 10.1186/1471-2202-16-S1-P131

**Published:** 2015-12-18

**Authors:** Ondrej Pokora, Lubomir Kostal

**Affiliations:** 1 Department of Mathematics and Statistics, Faculty of Science, Masaryk University, Brno, Czech Republic; 2 Institute of Physiology, Academy of Sciences of the Czech Republic, Prague, Czech Republic

## 

We address the problem of non-parametric estimation of the probability density function as a description of the probability distribution of noncorrelated interspike intervals (ISI) in records of neuronal activity. We also continue our previous effort [1,2] to propose alternative estimators of the variability measures. Kernel density estimators are probably the most frequently used non-parametric estimators of the probability distribution. However, there are also other non-parametric approaches. We focus on non-parametric methods based on a principle of extrema of the Fisher information. Specifically, we focus on the maximum penalized likelihood estimation of the probability density function proposed by Good and Gaskins [3], which can be understood as a kernel estimator with a particular kernel function [4]. Other non-parametric approach we would like to address is the spline interpolation proposed by Huber [5] which can uniquely estimate the ISI distribution.
